# Behavioural markers for autism in infancy: Scores on the Autism Observational Scale for Infants in a prospective study of at-risk siblings

**DOI:** 10.1016/j.infbeh.2014.12.017

**Published:** 2015-02

**Authors:** Isobel Gammer, Rachael Bedford, Mayada Elsabbagh, Holly Garwood, Greg Pasco, Leslie Tucker, Agnes Volein, Mark H. Johnson, Tony Charman, The BASIS Team

**Affiliations:** a King's College London, Institute of Psychiatry, Psychology & Neuroscience, Department of Psychology, Box PO77, Henry Wellcome Building, De Crespigny Park, Denmark Hill, London SE5 8AF, UK; b King's College London, Institute of Psychiatry, Psychology & Neuroscience, Department of Biostatistics, London, UK; c McGill University, Montreal, QC, Canada; d Centre for Brain and Cognitive Development, Birkbeck, University of London, London, UK

**Keywords:** Autism, ASD, Autism observation scale for infants (AOSI), Early behavioural markers, High-risk siblings

## Abstract

We investigated early behavioural markers of autism spectrum disorder (ASD) using the Autism Observational Scale for Infants (AOSI) in a prospective familial high-risk (HR) sample of infant siblings (*N* = 54) and low-risk (LR) controls (*N* = 50). The AOSI was completed at 7 and 14 month infant visits and children were seen again at age 24 and 36 months. Diagnostic outcome of ASD (HR-ASD) versus no ASD (HR-No ASD) was determined for the HR sample at the latter timepoint. The HR group scored higher than the LR group at 7 months and marginally but non-significantly higher than the LR group at 14 months, although these differences did not remain when verbal and nonverbal developmental level were covaried. The HR-ASD outcome group had higher AOSI scores than the LR group at 14 months but not 7 months, even when developmental level was taken into account. The HR-No ASD outcome group had scores intermediate between the HR-ASD and LR groups. At both timepoints a few individual items were higher in the HR-ASD and HR-No ASD outcome groups compared to the LR group and these included both social (e.g. orienting to name) and non-social (e.g. visual tracking) behaviours. AOSI scores at 14 months but not at 7 months were moderately correlated with later scores on the autism diagnostic observation schedule (ADOS) suggesting continuity of autistic-like behavioural atypicality but only from the second and not first year of life. The scores of HR siblings who did not go on to have ASD were intermediate between the HR-ASD outcome and LR groups, consistent with the notion of a broader autism phenotype.

## Highlights

• Behavioural atypicalities in emergent ASD in infancy include both social and non-social behaviours.
• Some of these atypicalities are found in HR siblings who do not go on to have ASD, supporting the notion of an early broader autism phenotype.
• Understanding the interplay between different neurodevelopmental domains across the first years of life is important to understand developmental mechanisms and to develop early interventions.

## 1 Introduction

Younger siblings of children with an autism spectrum disorder (ASD) represent a high-risk group for ASD, with recent estimates of the recurrence rate in siblings as high as 18.7% (Ozonoff et al., 2011). This allows prospective study of development from the first few months of life in infants who will later go on to receive a diagnosis of ASD. Within such prospective infant sibling designs, identifying the earliest differences or markers in those who go on to develop autism is a research priority (Zwaigenbaum, Bryson, & Garon, 2013). Current aetiological models of autism propose that typical developmental trajectories are derailed by complex interactions between underlying genetic and neurological vulnerabilities, environment and behaviour, with cascading developmental effects (Gliga, Jones, Bedford, Charman, & Johnson, 2014). However, the details of these developmental processes are poorly understood. It is hoped that understanding the ordering and interactive influences of the earliest biological and behavioural perturbations will elucidate developmental *mechanisms* that lead to the pattern of symptoms and impairments that characterise the clinical phenotype, as well as protective mechanisms that differentiate those at familial risk who go on to have non-ASD outcomes. This may in turn point to targets for treatment as well as improving identification of those infants at highest risk for the disorder in infancy, allowing very early intervention to be put in place (Green et al., 2013, in press).

### 1.1 Early behavioural signs of autism in high-risk studies

Recent reviews of high-risk (HR) sibling studies find convergent evidence for the emergence of overt behavioural markers between 12 and 18 months of age that distinguish, at a group level, those infants who go on to receive an ASD diagnosis from other HR infants and low-risk (LR) groups (Jones, Gliga, Bedford, Charman, & Johnson, 2014; Zwaigenbaum et al., 2013). At this age, clinically relevant behavioural differences span both social communication (e.g. gaze following (Bedford et al., 2012); social referencing (Cornew, Dobkins, Akshoomoff, McCleery, & Carver, 2012)) and stereotyped/repetitive behaviour (e.g. repetitive play (Christensen et al., 2010); repetitive movement (Loh et al., 2007) domains, as well as motor (Flanagan et al., 2012) and attentional atypicalities (Elsabbagh et al., 2013), appearing to represent early manifestations of later ASD symptoms. However, there is considerable heterogeneity at the individual level in the pattern of symptom emergence. Prior to 12 months of age, however, few overt behavioural markers for autism have been identified (Jones et al., 2014). In a recent report, Jones and Klin (2013) found that in a small sample of HR infants who went on to an ASD diagnosis, fixation on the eyes declined between 2 and 6 months of age. Other behavioural signs in the first year of life have included reduced gaze to people (Chawarska, Macari, & Shic, 2013) and vocal atypicalities (Paul, Fuerst, Ramsay, Chawarska, & Klin, 2010). Experimental studies have detected atypical neural response to social stimuli such as dynamic eye gaze from as young as 6 months of age (Elsabbagh et al., 2012).

### 1.2 The Autism Observation Scale for Infants (AOSI)

Whilst the research reviewed above and elsewhere (Gliga et al., 2014; Jones et al., 2014; Zwaigenbaum et al., 2013) has mostly used experimental tasks/paradigms and observational and parent-report methods, there is also a clinical need for an instrument that allows systematic observation of early-emerging atypicalities in infants at-risk for ASD. The Autism Observational Scale for Infants (AOSI; Bryson, Zwaigenbaum, McDermott, Rombough, & Brian, 2007) is a semi-structured, experimenter-led behavioural assessment designed to measure early behavioural markers of ASD in infants aged between 6 and 18 months. These include atypicalities or delays in social communication behaviours (e.g. anticipatory social response, social babbling, orientation to name, eye contact) and non-social behaviours (e.g. disengagement of visual attention, motor control and behaviour, atypical sensory behaviours) as well as aspects of temperament (e.g. reactivity, ease of transitions between activities).

Preliminary findings from the instrument's authors’ HR sibling cohort suggested that AOSI scores by 12 months but not at 6 months were promising as a predictor of later ASD outcomes based on 24 month ADOS classification (Zwaigenbaum et al., 2005). The same group have reported that whilst many individual AOSI items across domains (e.g. orients to name, eye contact, reactivity) at both age 6 and 18 months differentiated the group of HR siblings who go on to have an ASD classification at 36 months and LR controls, only atypical motor behaviour differentiated HR siblings who go on to have ASD from HR siblings who do not at both timepoints (Brian et al., 2008, 2013). Early behavioural atypicalities measured by the AOSI at 12 months have also been shown to characterise nearly one fifth of HR siblings who do not go on to have ASD (Georgiades et al., 2013), consistent with the notion of sub-clinical manifestations of ASD being present at an enhanced rate in family members of individuals with ASD, referred to as the broader autism phenotype (BAP; Bolton et al., 1994).

### 1.3 The current study

The present study sought to replicate in an independent sample whether predictive associations exist between AOSI scores in early (at around 7 months) and later infancy (around 14 months) and ASD outcome at 36 months. In the present study we analysed AOSI data from both 7 and 14 month timepoints in a cohort of HR siblings and LR controls subsequently followed up at 24 and 36 months to answer the following questions:

1. Do scores on the AOSI differ between HR siblings and LR controls at 7 and 14 months?
2. Do scores on the AOSI differ between those HR siblings who go on to have a diagnosis of ASD from those HR siblings who do not?
3. Within the HR group are there associations between AOSI scores at the 7 and 14 month timepoint and later scores on the Autism Diagnostic Observational Schedule (ADOS-G; Lord et al., 1999) at 24 and 36 months?

## 2 Methods

Ethical approval for the BASIS study was obtained from NHS NRES London REC (08/H0718/76). One or both parents gave informed, written consent for their child to participate.

### 2.1 Participants

One hundred and four children (54 HR, 50 LR) were recruited as part of the British Autism Study of Infant Siblings (BASIS; www.basisnetwork.org). They were seen on four visits when aged 6–10 months (mean = 7.35, SD = 1.21; hereafter 7 m), 11–18 months (mean = 13.79, SD = 1.46; hereafter 14 m), and then around their 2nd birthday (mean = 23.9 months, SD = .95; hereafter 24 m), and third birthday (mean = 37.93 months, SD = 3.02; hereafter 36 m).

Each HR infant had an older sibling (in 4 cases, a half-sibling) with a community clinical ASD diagnosis (hereafter, proband), confirmed on the basis of information in the Development and Wellbeing Assessment (DAWBA; Goodman, Ford and Richards 2000) and the Social Communication Questionnaire (SCQ; Rutter, Bailey and Lord 2003) by expert clinicians on our team (TC, PB). Most probands met ASD criteria on both measures (*n* = 44). While a small number scored below threshold on the SCQ (*n* = 4), no exclusions were made due to meeting the DAWBA threshold and expert opinion. For two probands, data were only available on one measure, and for four probands, neither measure was available (aside from parent-confirmed local clinical diagnosis). Parent-reported family medical histories were examined for significant conditions in the proband or extended family members (e.g., Fragile X syndrome, tuberous sclerosis) with no such exclusions deemed necessary.

LR controls were full-term infants (gestational ages 37 to 42 weeks; 3 born 32 to 36 weeks) recruited from a volunteer database at the Birkbeck Centre for Brain and Cognitive Development. Medical history review confirmed lack of ASD within first-degree relatives. All LR infants had at least one older sibling (in three cases, only half-siblings). The SCQ was used to confirm absence of ASD in these older siblings, with no child scoring above instrument cut-off (≥15; *n* = 1 missing data).

### 2.2 The Autism Observational Scale for Infants (AOSI)

The Autism Observation Scale for Infants (AOSI; Bryson et al., 2007; revised version used in this study; Brian et al*.*, 2008) is an experimenter-led, semi-structured observational assessment, developed to study the nature and emergence of ASD-related behavioural markers in infancy (6–18 months). A standard set of objects and toys are used across five activities – each with a specified series of presses for a particular behaviour – and two periods of free-play. Responses to presses and observations made throughout the assessment are used to code nineteen items (see Brian et al., 2008; Bryson et al., 2007; for full description of presses, items and coding; items listed in the Appendix). Each item is coded on a scale from 0 to 2 or 0 to 3. A rating of 0 denotes typical behaviour and higher scores denote increasing atypicality. In the current study the 19 item version of the AOSI reported by Brian et al. (2008) was used. The AOSI yields a Total Score (sum of all codes; max score 44). The AOSI is administered by a trained examiner who sits at a table opposite the infant who is held on the parent's lap. AOSIs were administered by research-reliable research staff and the majority of administrations were double-coded by the examiner and an observer. Agreement between the two coders was excellent at both 7 months (*n* = 92, intraclass correlation coefficient = .97) and 14 months (*n* = 85, intraclass correlation coefficient = .95). When codes differed between researchers, they discussed and agreed on a consensus code, where no observer codes were available the examiner's code was used.

### 2.3 Developmental assessments and outcome groups

All participants were assessed at all visits on the Mullen Scales of Early Learning (MSEL; Mullen, 1995)—a measure of developmental abilities yielding an Early Learning Composite (ELC) standardised score (mean = 100; SD = 15). In order to explore the association between verbal and nonverbal developmental abilities and AOSI scores we calculated mean *T*-scores from the two Verbal (Receptive Language, Expressive Language) and two Nonverbal (Fine Motor, Visual Reception) Mullen subscales.

Of the 54 HR infants recruited, 53 were retained to the 36 m visit when comprehensive diagnostic assessment was undertaken. At 36 m parents of HR siblings completed the Autism Diagnostic Interview—Revised (ADI-R; Lord, Rutter, & Le Couteur, 1994) and the SCQ (Rutter et al., 2003), and both HR and LR toddlers were assessed with the Autism Diagnostic Observation Schedule (ADOS-G: Lord et al., 1999; 24 m module 1*N* = 50, module 2*N* = 2; 36 m module 2*N* = 50 toddlers, module 1*N* = 3) and the revised Social Affect and Repetitive and Restrictive Behaviours subtotal and calibrated severity scores computed (Gotham et al., 2007). Assessors were not blind to risk-group status. Assessments were conducted by or under the close supervision of clinical researchers (i.e., psychologists, speech therapists) with demonstrated research-level reliability. Different teams of researchers saw participants at the first two visits and the second two visits. Those assessing developmental outcomes were blind to infants’ performance on the AOSI.

In determining diagnostic outcome status, four clinical researchers (KH, SC, GP, TC) reviewed information across the 24 m (including an ADOS-G assessment for the HR siblings) and 36 m visit (including both ADOS-G and ADI-R administration for the HR siblings). Seventeen toddlers (11 boys, 6 girls) met ICD-10 (World Health Organisation, 1993) criteria for an ASD (combining ICD-10 childhood autism and pervasive developmental disorder (hereafter, HR-ASD subgroup)). The remaining 36 toddlers (10 boys, 26 girls) did not meet diagnostic criteria for ASD (HR-No ASD). For the LR control group, in the absence of a full developmental history (no ADI-R was administered) no formal clinical diagnoses were assigned but none had a community clinical ASD diagnosis at 36 months.

It is worth noting that the recurrence rate reported in the current study (32.1%) is higher than that reported in the large consortium paper published by Ozonoff and colleagues (18.7%; Ozonoff et al., 2011). This is likely to reflect the modest size at-risk sample in the current study (*N* = 53). Whilst recurrence rates approaching 30% have been found in other moderate size samples (Landa & Garrett-Mayer, 2006; Paul et al., 2010) these rates are sample specific and will likely not be generalizable as findings from larger samples where autism recurrence rates converge between 10% and 20% (Constantino et al., 2010; Sandin et al., 2014). Similar procedures combining all information from standard diagnostic measures and clinical observation and arriving at a ‘clinical best estimate’ ICD-10 diagnosis was used in the present study in line with other familial at-risk studies and was conducted by an experienced group of clinical researchers.

HR siblings and LR controls did not significantly differ from each other in age at any visit (*t*-tests, all *p*s > .34), nor did the HR outcome groups or LR controls differ from one another in age at any visit (all *p*s > .20; see Table 1 for descriptive statistics on all measures). Whilst the ELC scores of the HR siblings were in the average range, at each visit their scores were lower than those for the LR controls (*t*-tests, all *p*s < .01). The HR-ASD group had lower ELC scores than the LR group at all four visits (all *p*s < .05) and lower ELC scores compared to the HR-No ASD group at the 14 m and 36 m visits (both *p* < .05) but not the 7 m and 24 m visits.

### 2.4 Analysis

Due to the skewed distribution of the AOSI Total Score a square root transformation was applied and the transformed data met assumptions of normality, with the exception of the LR group at 14 m of age (*p* < .05). HR versus LR scores were compared using ANOVA and HR-ASD, HR-No ASD and LR scores were compared using a one-way ANOVA and post-hoc Tukey HSD tests. Cohen's d effect sizes are reported (Cohen, 1988). Following this in order to control for verbal and nonverbal developmental level the Mullen mean Verbal and Nonverbal *T*-scores were covaried and post-hoc least significant difference (LSD) tests conducted. For individual AOSI items, HR versus LR scores were compared using Mann–Whitney tests and HR-ASD, HR-No ASD and LR scores were compared using Kruskal–Wallace tests and significant differences followed-up using post-hoc Mann–Whitney tests. Given the larger number of items but also allowing for the exploratory nature of the analysis, a moderately conservative significance level of *p* < .01 was used. Correlations between AOSI scores and the total scores on the ADOS (Social Affect and Restricted and Repetitive Behaviour subtotals combined) at 24 months and 36 months in the HR group only were examined using Pearson's product moment correlations.

## 3 Results

### 3.1 HR versus LR group differences

As shown in Table 2, at 7 m the HR group had a higher AOSI score than the LR group *F*(1, 102) = 4.10, *p* < .045 (*d* = .39). At 14 m the HR group had a higher AOSI score than the LR group but the difference was not statistically significant *F*(1, 99) = 3.36, *p* = .07 (*d* = .36). However, when Mullen Verbal and Nonverbal *T*-score at each age point was covaried, the difference between the HR and LR groups was no longer significant (7 m: *F*(3, 99) = .66, *p* = .37; 14 m: *F*(3, 96) = 2.24, *p* = .14). At 7 m but not 14 m the covariate effect for Mullen Verbal *T*-score was significant (*F*(3, 99) = 4.48, *p* < .05).

### 3.2 HR outcome group differences

**Fig. 1:**
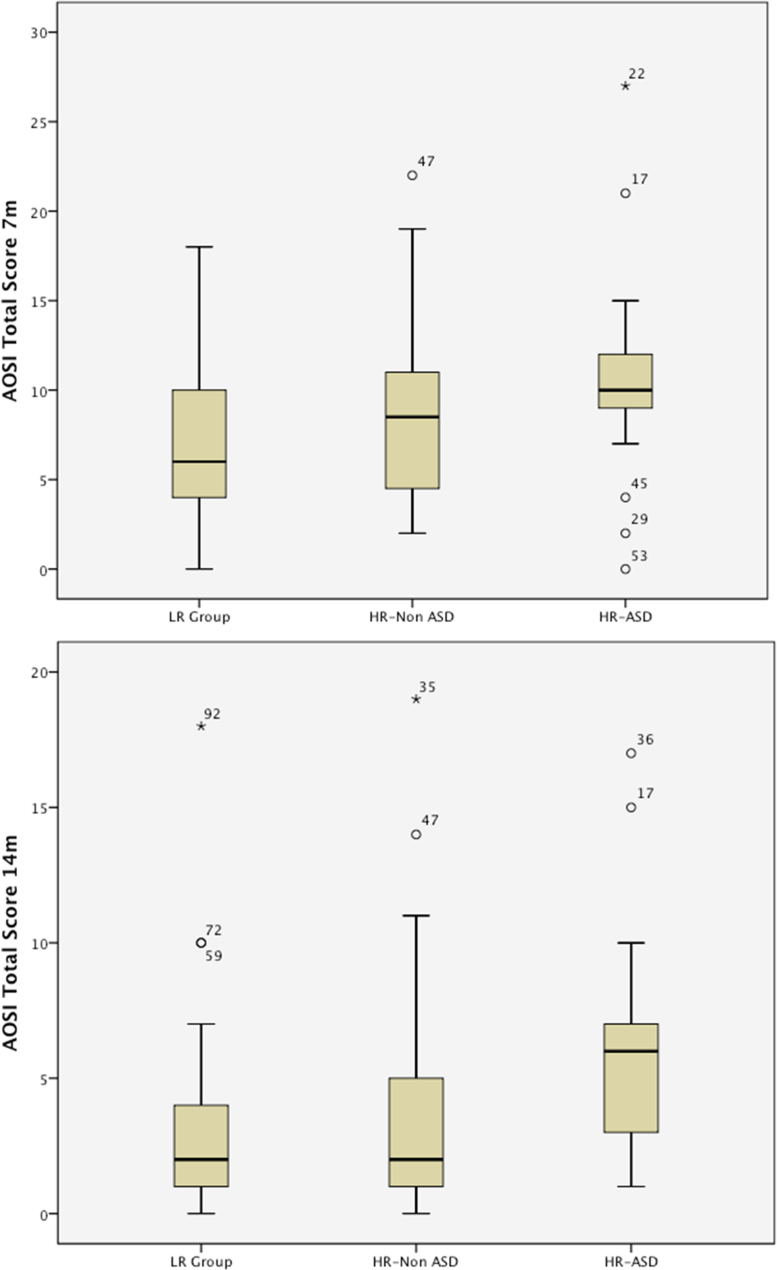
Boxplots of the AOSI total scores by outcome groups at 7 m and 14 m^1^.

Comparing AOSI scores for the HR-ASD and HR-No ASD outcome groups and the LR group, at 7 m the one-way ANOVA just missed significance *F*(2, 98) = 2.94, *p* = .058 (see Table 2 and Fig. 1). At 14 m, the three outcome group comparison was significant *F*(2, 96) = 4.43, *p* = .014. Post-hoc Tukey tests showed that the HR-ASD group scored higher than the LR group (*p* = .01; *d* = .81). The HR-ASD group also had a marginally but not significantly higher AOSI score than the HR-No ASD group (*p* = .07; *d* = .65). These analyses were repeated covarying for Mullen Verbal and Nonverbal *T*-score at each age. The HR outcome groups and LR group did not differ from each other at 7 m of age, *F*(4, 95) = .61, *p* = .47 but did differ from each other at 14 m of age, *F*(4, 93) = 3.85, *p* = .03. Post-hoc LSD tests showed that at 14 m the HR-ASD group scored higher than both the LR group (*p* *<* .01) and the HR-No ASD group (*p* = .03) and that the HR-No ASD group and LR group did not differ from each other (*p* = .53). At 7 m but not 14 m the covariate effect for Mullen Verbal *T*-score was significant (*F*(4, 95) = 4.31, *p* < .05).

### 3.3 Individual AOSI items

In terms of individual items, at 7 m the HR and LR groups did not differ on any items but the HR group scored higher than the LR controls on one item at 14 m (orientation to name; *U* = 931.00, *p* < .01). In terms of HR-ASD vs. HR-No ASD outcome groups vs. LR group differences, at 7 m there were significant differences for the following items: visual tracking (*χ*^2^ = 9.99, *p* < .01; HR-No ASD > LR: *U* = 634.50, *p* < .01) and Social Referencing (*χ*^2^ = 10.78, *p* < .01; HR-ASD > LR: *U* = 206.50, *p* < .01). At 14 m there were significant differences for the following items: orientation to name (*χ*^2^ = 11.50, *p* < .01; HR-No ASD > LR: *U* = 596.00, *p* < .01; HR-ASD > LR: *U* = 282.50, *p* < .01), Engagement of Attention (*χ*^2^ = 9.75, *p* < .01; no significant post-hoc tests) and Social Referencing (*χ*^2^ = 9.75, *p* < .01; no significant post-hoc tests).

### 3.4 Associations between AOSI scores at 7 m and 14 m and ADOS scores at 24 m and 36 m

To examine associations over time between behavioural atypicality as measured by the AOSI in infancy and the ADOS in toddlerhood correlations were examined in the HR group. Since the AOSI measures attentional disengagement and atypical motor behaviours, as well early social communication behaviours, AOSI scores were compared to the ADOS ‘total score’. AOSI score at 7 m was not associated with ADOS score at either 24 m (*r* = .23, *p* = .10) or 36 m (r = .16, p = .27). However, AOSI score at 14 m was significantly associated both with ADOS score at 24 m (*r* = .30, *p* = .03) and at 36 m (*r* = .38, *p* = .005).

## 4 Discussion

Consistent with previous reports (Brian et al., 2008, 2013; Georgiades et al., 2013) behavioural atypicalities as measured by the AOSI differentiated the HR and LR groups in the first year (7 months) and (marginally) early in the second year (14 months) of life. At 14 months (but not 7 months) these behavioural markers also discriminated between those HR infants who went on to an ASD diagnosis and LR controls and marginally between HR who did and did not go on to an ASD diagnosis. The HR versus LR group comparisons were attenuated when verbal and nonverbal developmental level was controlled but the HR-ASD outcome group differences remained significant. The HR-No ASD outcome group scored intermediate between the HR-ASD outcome group and the LR control group.

Several aspects of this pattern of differences are worthy of comment. First, the HR versus LR group comparisons are modest only in effect size (.39 at 7 months and .36 at 14 months). However, in those HR infants who went on to meet diagnostic criteria for ASD at 36 months by the 14 month timepoint the effect sizes were large (.81 compared to the LR controls; .65 compared to HR infants who did not go on to meet criteria for ASD at 36 months. The current sample is modest in size but, notwithstanding this, significant sub-group differences were still found because of the relatively large behavioural differences on this observational measure of early autistic atypicality. Although the AOSI combines developmental abilities (e.g. imitation) and the presence of atypical or unusual behaviours (e.g. atypical motor and sensory behaviours) the effects of developmental level as measured by the Mullen verbal and nonverbal subscales did not predominate, with only verbal ability at 7 month being significantly associated with the AOSI total score. Finally, the HR-No ASD group performed intermediate between the HR-ASD group and LR group, both on overall total scores (Table 2) and on individual items (see below). The boxplots in Fig. 1 show that the interquartile range of this group span across those of both the HR-ASD and LR groups, suggesting that characteristics that might be considered as aspect of the early ‘broader autism phenotype’ (BAP) are seen in some but not all of the infants at familial high-risk who do not go onto an ASD presentation at 36 months of age (see also Georgiades et al., 2013).

### 4.1 Individual items

Although the initial report on the AOSI found that scores were predictive of a diagnosis at 12 months but not 6 months (Zwaigenbaum et al., 2005), subsequent studies have found that some behaviours at 6 months differentiate HR siblings and LR controls (e.g. eye contact, social referencing) and also those HR siblings who go on to have an ASD diagnosis from both LR controls (e.g. reactivity, disengagement of attention) and HR siblings who do not go on to have an ASD (e.g. atypical motor control) (Brian et al., 2013). In terms of individual AOSI items we found no HR vs. LR group differences at 7 m and HR siblings only scored higher than LR controls on one item at 14 m (orientation to name). We found that social referencing at 7 m and orientation to name also at 14 m differentiated the HR siblings who went on to have an ASD from LR controls. Furthermore, in line with the BAP concept (Georgiades et al., 2013; Ozonoff et al., 2014), HR siblings who did not go on to have an ASD also showed higher levels of atypical behaviour at 7 m (visual tracking) and 14 m (orientation to name), compared to LR controls. However, these item-level findings should be considered exploratory given that a strict correction for multiple testing was not undertaken and require replication in other samples.

In contrast to previous reports (Brian et al., 2008, 2013; Georgiades et al., 2013), we conservatively adjusted for the differences in verbal and non-verbal developmental ability between the groups. It is increasingly apparent, consistent with the phenotype of ASD, that both developmental and language delays are part of the BAP at a group level (Messinger et al., 2013; Ozonoff et al., 2014) and, whilst covarying for these differences one might be taking out some of the variance of interest, early atypical behaviours still discriminated between the infants who went on to have ASD and those who did not.

### 4.2 Associations between the AOSI and later ADOS scores

Within the HR sibling group we examined the association between early behavioural atypicalities as measured by the AOSI and later early symptoms of autism as measured by the ADOS. AOSI scores at 7 months were not associated with later ADOS scores but AOSI scores at 14 months were moderately associated with ADOS scores at 24 and 36 months. Although the two instruments are not identical there is a considerable overlap in the concepts, behaviours and scoring systems, and this suggests a moderate degree of continuity of autistic-like behavioural atypicality from the beginning of the second year of life into the toddler years. However, as has been found with many experimental measures (Jones et al., 2014), with a few exceptions, this continuity is not apparent from as early as 6 to 8 months of age. As such, it appears that the AOSI is successfully capturing very early emerging autistic behaviours. Larger samples will be required in order to test both how predictive such early behavioural markers are at an individual, as opposed to a group, level and in order to trace the longitudinal trajectory of the emergence of such behaviours over this early time course (Landa et al., 2012, 2013). This work is much needed as increasingly in some communities concerns about possible autism are raised about some children in the second year of life, in particular younger siblings of a child with an ASD given the now well-established recurrence rate of between 10% and 20% (Constantino et al., 2010; Ozonoff et al., 2011; Sandin et al., 2014).

### 4.3 Limitations

We consider these findings preliminary due to the modest sample size and they will require confirmation in larger and other independent samples. However, it is the first independent report on the AOSI and replicates some of the findings from the instrument's originators (Brian et al., 2008, 2013; Zwaigenbaum et al., 2005). We also note several limitations in the design, including non-blind assessment (to risk status) at both the infancy and toddler assessments, although the team conducting the toddler visits were blind to infant AOSI scores.

## 5 Conclusions

The current findings confirm the emerging picture that early behavioural atypicalities in emergent ASD include both social and non-social behaviours (see Jones et al., 2014; for a review). Some of these atypicalities are found only in HR siblings who go on to have ASD but others are also found in HR siblings who do not, supporting the notion of an early broader autism phenotype (Georgiades et al., 2013; Ozonoff et al., 2014). Understanding the interplay between different neurodevelopmental domains across the first years of life and the influences on these will be important both to understand the developmental mechanisms that lead to the ASD behavioural phenotype and to inform approaches to developing early interventions (Green et al., 2013, in press; Wallace & Rogers, 2010).

## Figures and Tables

**Table:** 

1. Visual tracking
2. Disengagement of attention
3. Orientation to name
4. Differential response to facial emotion
5. Anticipatory response
6. Imitation of actions
7. Social babbling
8. Eye contact
9. Reciprocal social smile
10. Coordination of eye gaze and action
11. Reactivity
12. Cuddliness [not included in this version]
13. Soothability [not included in this version]
14. Social interest and shared affect
15. Transitions
16. Motor control and behaviour
17. Atypical motor behaviours
18. Atypical sensory behaviours
19. Engagement of attention
20. Insistence on having particular object
21. Sharing interest

**Table 1:** Descriptives for high risk and low risk groups and high risk group by 36 month outcome.

	LR controls	HR sibs		
		Combined	HR-No ASD	HR-ASD
*N* (M, F)	*N* = 50 (21, 29)	*N* = 54 (21, 33)	*N* = 36 (10, 26)	*N* = 17 (11, 6)
	Mean (SD)	Mean (SD)	Mean (SD)	Mean (SD)
Age 7 m	7.38 (1.24)	7.31 (1.20)	7.17 (1.16)	7.53 (1.23)
Age 14 m	13.92 (1.33)	13.68 (1.57)	13.51 (1.24)	13.94 (1.60)
Age 24 m	23.87 (.68)	23.92 (1.15)	23.89 (1.15)	24.00 (.97)
Age 36 m	38.23 (3.05)	37.66 (2.99)	37.61 (3.36)	37.76 (2.11)
7 m ELC^1^	104.42 (11.31)^a^	94.00 (12.88)^a^	95.03 (10.69)	92.13 (17.30)^b^
14 m ELC^1^	106.11 (15.73)^a^	97.40 (17.91)^a^	102.11 (16.04)^c^	89.18 (18.30)^b^
24 m ELC^1^	116.02 (13.98)^a^	102.25 (19.77)^a^	104.25 (17.14)	97.75 (24.74)^b^
36 m ELC^1^	115.77 (16.25)^a^	105.38 (21.52)^a^	110.11 (15.87)^c^	94.75 (28.51)^b^
24 m ADOS CSS^2^	–	3.46 (2.32)	2.75 (1.92)	5.06 (2.41)
36 m ADOS CSS^2^	2.85 (1.88)	4.32 (2.62)	3.42 (2.34)	6.24 (2.14)
36 m ADI social^3^	–	4.54 (5.33)	2.22 (3.20)	9.75 (5.54)
36 m ADI communication^3^	–	4.44 (4.82)	2.67 (3.46)	8.44 (5.14)
36 m ADI repetitive^3^	–	1.60 (2.02)	.69 (1.06)	3.63 (2.22)

1 Mullen Scales of Early Learning Early Learning Composite Standard Score.

2 ADOS: Autism Diagnostic Observation Schedule Calibrated Severity Score.

3 ADI: Autism Diagnostic Interview.

a HR group < LR group, *p* < .01.

b HR-ASD group < LR group, *p* < .05.

c HR-ASD group < HR-No ASD group, *p* < .05.

**Table 2:** AOSI scores for high risk and low risk groups and high risk group by 36 month outcome.

	LR controls	HR sibs		
		Combined	HR-No ASD	HR-ASD
	*N* = 50	*N* = 54	*N* = 36	*N* = 17
7 m	7.12 (4.07)	9.33 (5.63)	8.83 (5.14)	10.82 (6.42)
14 m	3.17 (3.25)	4.64 (4.47)	3.97 (4.38)	6.18 (4.50)

## Footnotes

1 The thick band is the median, the edges of the box the upper and lower quartiles, the whiskers the highest and lowest values that are not outliers, the circles outliers between 1.5 SD and 3.0 SD and the stars outliers >3.0 SD from mean.
